# Supplementary material for: B1 oligomerization regulates PML nuclear body biogenesis and leukemogenesis
Source: Nat Commun. 2019 Aug 22;10:3789. doi: 10.1038/s41467-019-11746-0 (PMC6706441; doi:10.1038/s41467-019-11746-0)
Supplement: Supplementary file 1 — Supplementary Information [file 41467_2019_11746_MOESM1_ESM.pdf]

Supplementary Information

B1 oligomerization regulates PML nuclear body biogenesis and leukemogenesis

Y. Li, X. Ma, Z. Chen, H. Wu, P. Wang *et al.*

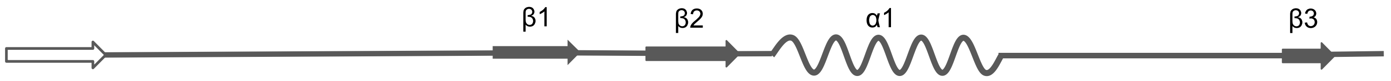


```
PML     120 RQIVDAQAVCTRCKE-----SADFWCFECEQLLCAKCFEA-HQWFLK----HEA---RPLAE 168
TRIM24      STVEKSNQVCTSCEDNA---EANGFCVECVEWLCKTCIRA-HQR-VKFTKDHTVRQKEEVSP
TRIM28      TDAQDANQCCTSCEDNA---PATSYCVECSEPLCETCVEA-HQR-VKYTKDHTVRSTGPA--
TRIM33      SSDEKSEQVCTSCEDNA---SAVGFCVECGEWLCKTCIEA-HQR-VKFTKDHLIRKKEDVS-
TRIM16      PAGEGKEVLCDFCLDDTRRVKAVKSCLTCMVNYCEEHLQP-HQVNIKLQS-HLLTEPVKDH-
TRIM45      ---EGQGLVCDLCND-R---EVEKRCQTCKANLCHFCCQA-HRR-QKKTTYHTMVDLKDLKG
TRIM18      TMTSAEKVLCQFCDQDP-AQDAVKTCVTCEVSYCDECLKATHPN-KKPFTGHRLIEPIPDSH
TRIM 1      TAMSSERIACQFCEQDP-PRDAVKTCITCEVSYCDRCLRATHPN-KKPFTSHRLVEPVPDTH
TRIM23      -ISGESIIRCDEDEAH----LASVYCTVCATHLCSECSQVTHS--TKTLAKHRRVPLADK--
TRIM71      ALLLRRPHGCSSCDEGN---AASSRCLDCQEHLCDNCVRA-HQR-VRLTKDHYIERGPPGPG
TRIM 9      -----AALKCQLCEKAP-K-EATVMCEQCDVFYCDPCRLRCHPP-RGPLAKHRLVPPAQGR-
TRIM67      TSAAAAVAICQLCDRTP-PEPAATLCEQCDVLYCSACQLKCHPS-RGPFAKHRLVQPPPPPP
TRIM36      ---AATAIMCDLCKPPPQESTKS--CMDCSASYCNECFKIHHPWGTIK-AQHEYVGPTTNFR
TRIM 8      ----PAALHCVFCRRGP-PLPAQKVCLRCEAPCCQSHVQT-HLQQPSTARGHLLVEAD----
TRIM42      FDRSSGPILCQVCRNKR---IAYKRCITCRLNLCNDCLKAFHS-DVAMQD-HVFVDTSAEE-
                     *  *            *  *    *  *    *         *
```

SD1 SD2

Supplementary Figure 1. Sequence alignment of TRIM B1-boxes. The Zn binding residues are in bold font and highlighted with “*” underneath the sequences. PML B1 SD1 and SD2 are bracketed. The structural determinants in PML B1 dimeric interfaces are colored in blue, purple and yellow, respectively. Of note, PML B1 is the only B1-box that displays oligomerization activity. The other B-box oligomerizations are mostly observed in B2-box (Figure 2b and Supplementary Figure 2b).

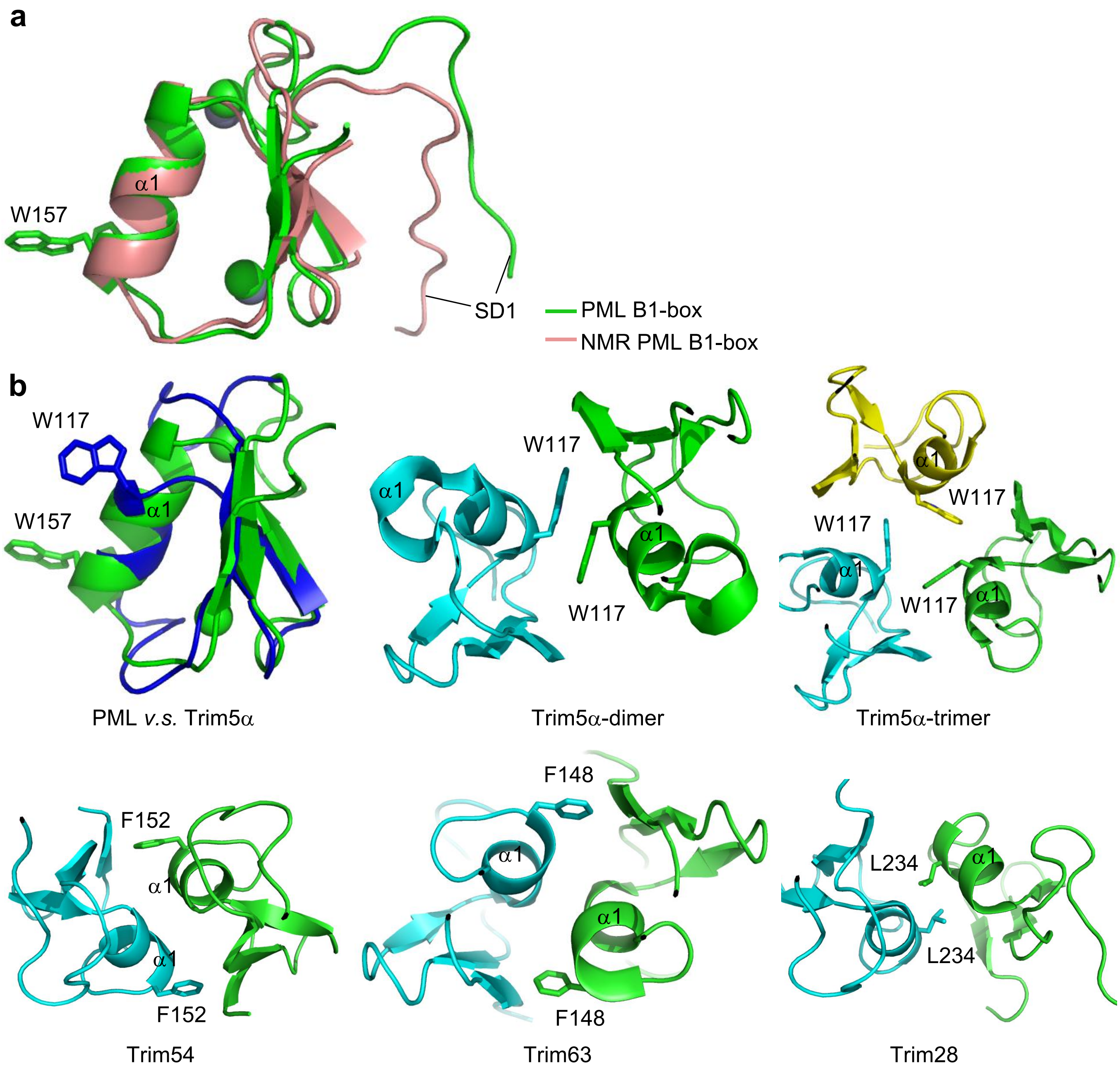

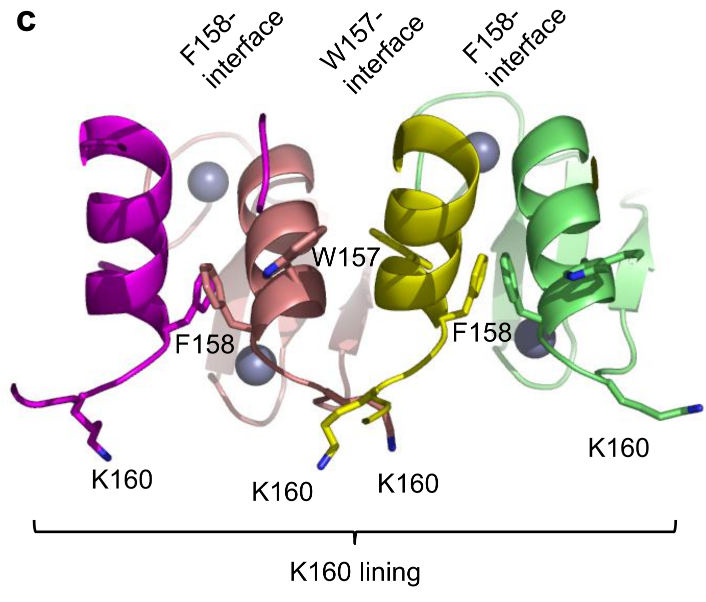

Supplementary Figure 2. **PML B1 and TRIM B-box oligomerization.** a) Structural superimposition between NMR (salmon) and crystallographic (green) PML B1s. W157 is shown in stick representation. SD1 is labelled. b) Structural comparison between PML B1-box and TRIM B-boxes. Top-left panel, superimposition between PML and TRIM5α. Top-middle panel, TRIM5α dimer. Top-right panel, TRIM5α trimer. Bottom left panel, TRIM54 dimer. Bottom-middle panel, TRIM63 dimer. Bottom-right panel, TRIM28 dimer. The bulky hydrophobic residues in the conserved α1 helices are shown in stick representation. c) Intermittent arrangement of W157- and F158-interfaces. K160, a key sumoylation site in PML and PML-RARα is highlighted with stick representation. The intermittent arrangements of W157- and F158-dimerization give rise to an interesting K160 lining. The local concentration effect might explain why this residue, not K65 or K490, is engaged in SUMO chain elongation[1].

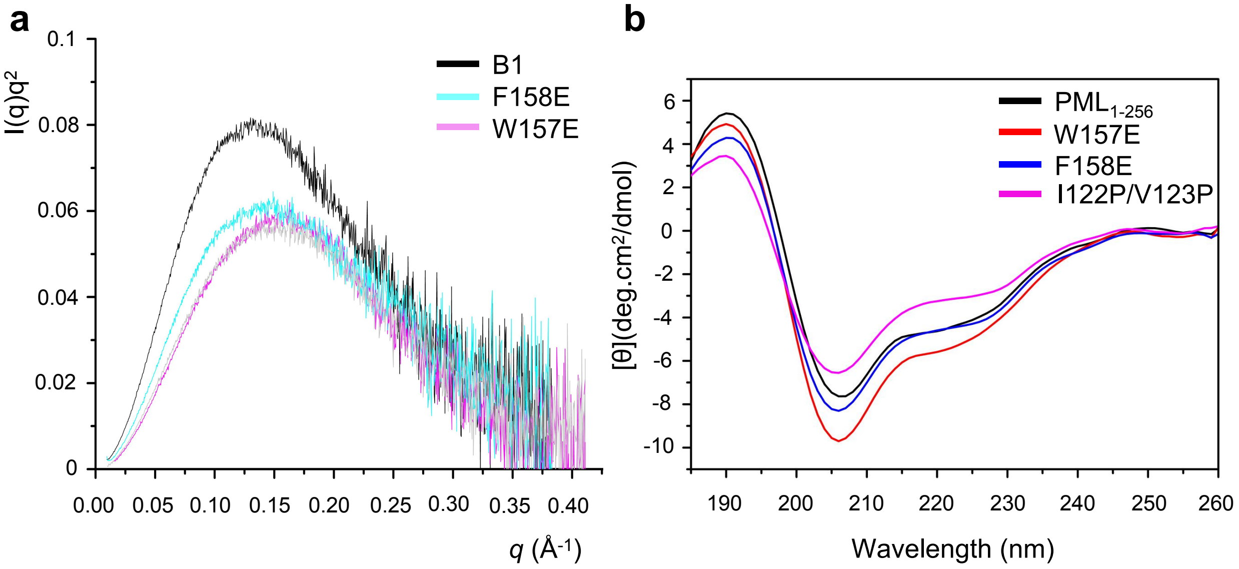

Estimate of protein secondary structure

	PML_{1-256}	W157E	F158E	I122P/V123P
Wavelength(nm)	185-260	185-260	185-260	185-260
Helix	25.3%	24%	24%	22.8%
Antiparallel	7.7%	7.5%	7.8%	8.4%
Parallel	5.2%	4.6%	4.5%	4.4%
Beta-Turn	29.3%	32.1%	32.8%	34.5%
Rndm.Coil	34.5%	35.2%	34.3%	34.3%
Total Sum	102%	103.4%	103.5%	104.4%

Supplementary Figure 3. **Biophysical characterization of PML B1 and PML_{1-256}.** a) Kratky plots of PML B1 and mutants. b) CD spectroscopy of PML_{1-256} and mutants. The spectra were the average of not less than three scans and presented as mean residue molar ellipticity [θ] (deg.cm^{2}dmol^{-1}). Source data are provided as a source data file. The secondary structure contents of WT and mutants were estimated using circular dichroism deconvolution program CDNN.

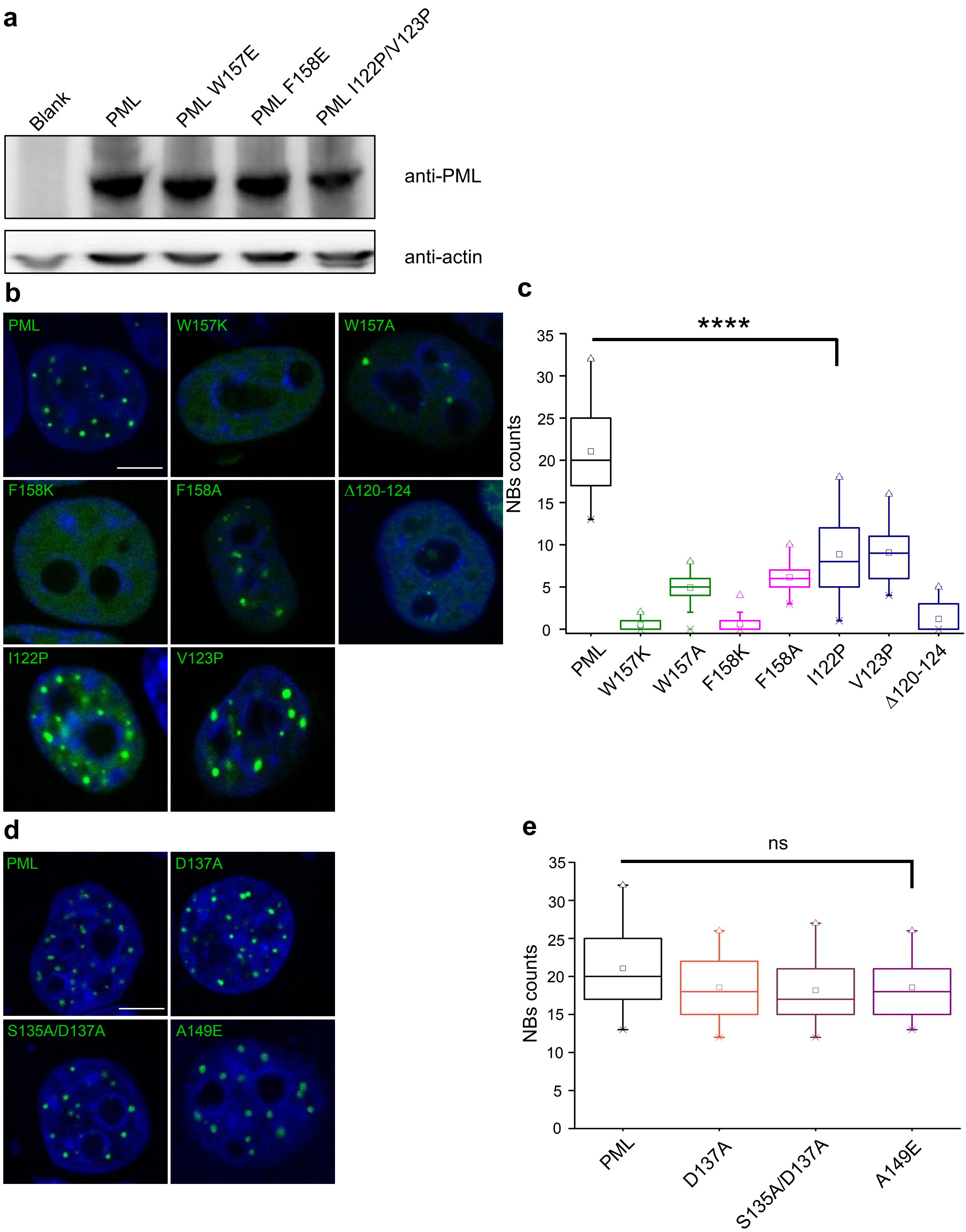

Supplementary Figure 4. More evidence on B1 oligomerization. a) The PML expression levels in HeLa$^{Pml-/-}$ cells. The Western blot and antibody against PML (Abcam) were used to monitor the WT and mutant expressions in HeLa$^{Pml-/-}$ cells. Source data are provided as a Source Data file. b, c) PML NB formation precluded by B1 mutants that targeting W157-, F158- and SD1-interfaces.Scar bar is 5 μm. Values are means ± S.E. ****, $p < 0.0001$. Source data are provided as a Source Data file. d, e) In marked contrast, the mutation targeting NMR B1 dimeric interface had little impact on NB biogenesis.Scar bar is 5 μm. Altogether, the results presented here favour the crystallographic oligomerization, but not the NMR dimerization[2]. ns, no statistical significance. All experiments have been done at least >6 independent replicates. Source data are provided as a Source Data file.

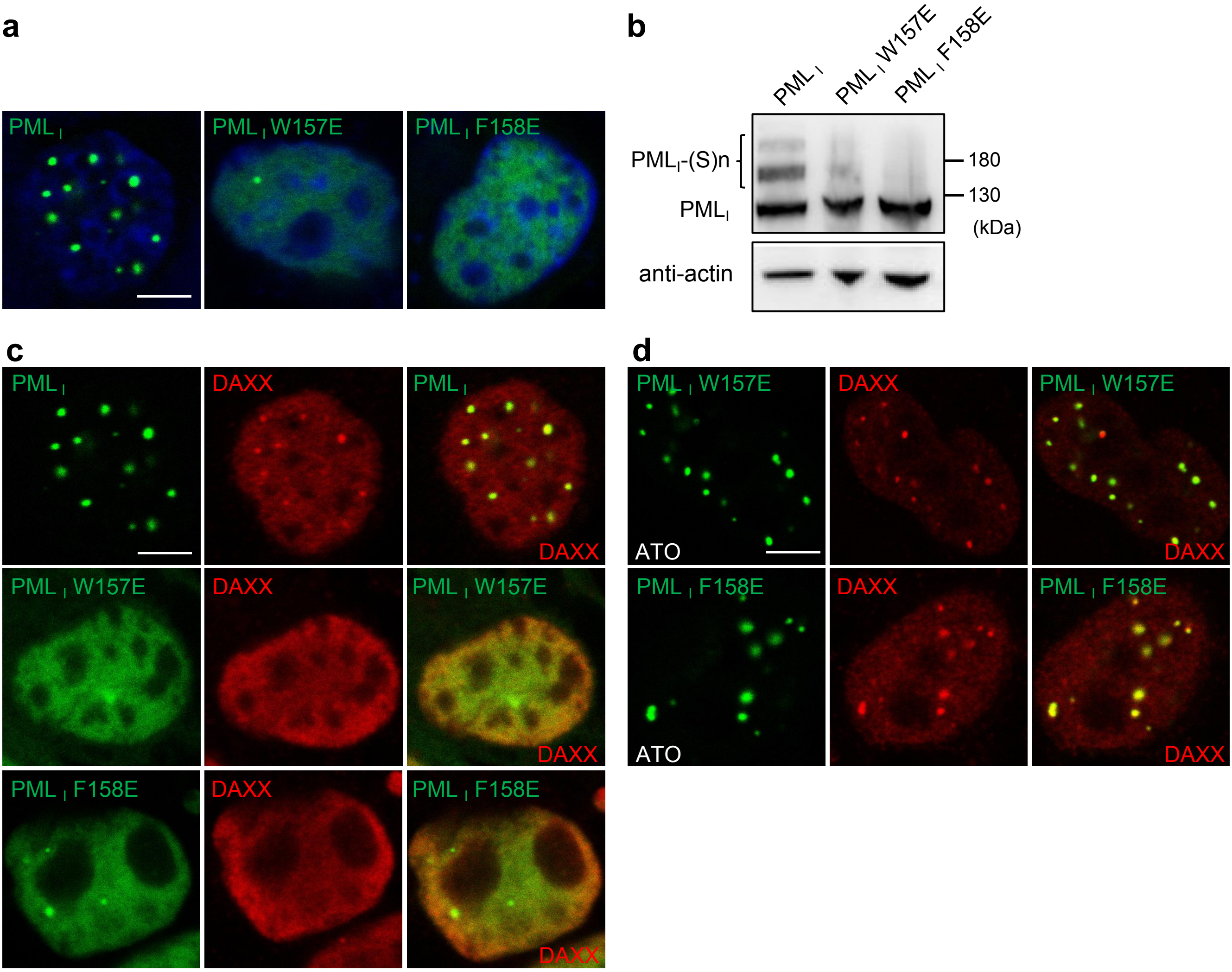

Supplementary Figure 5. More experimental results repeated in PML isoform I, termed PML$_I$. a) PML$_I$ nuclear body formation assay.Scar bar is 5 µm. b) PML$_I$ sumoylation at basal level. Source data are provided as a source data file. c) Immuno co-localization between PML$_I$ and DAXX. Scar bar is 5 µm. d) ATO could rescue B1 mutants in terms of PML$_I$ NB biogenesis and partners recruitment. Scar bar is 5 µm.

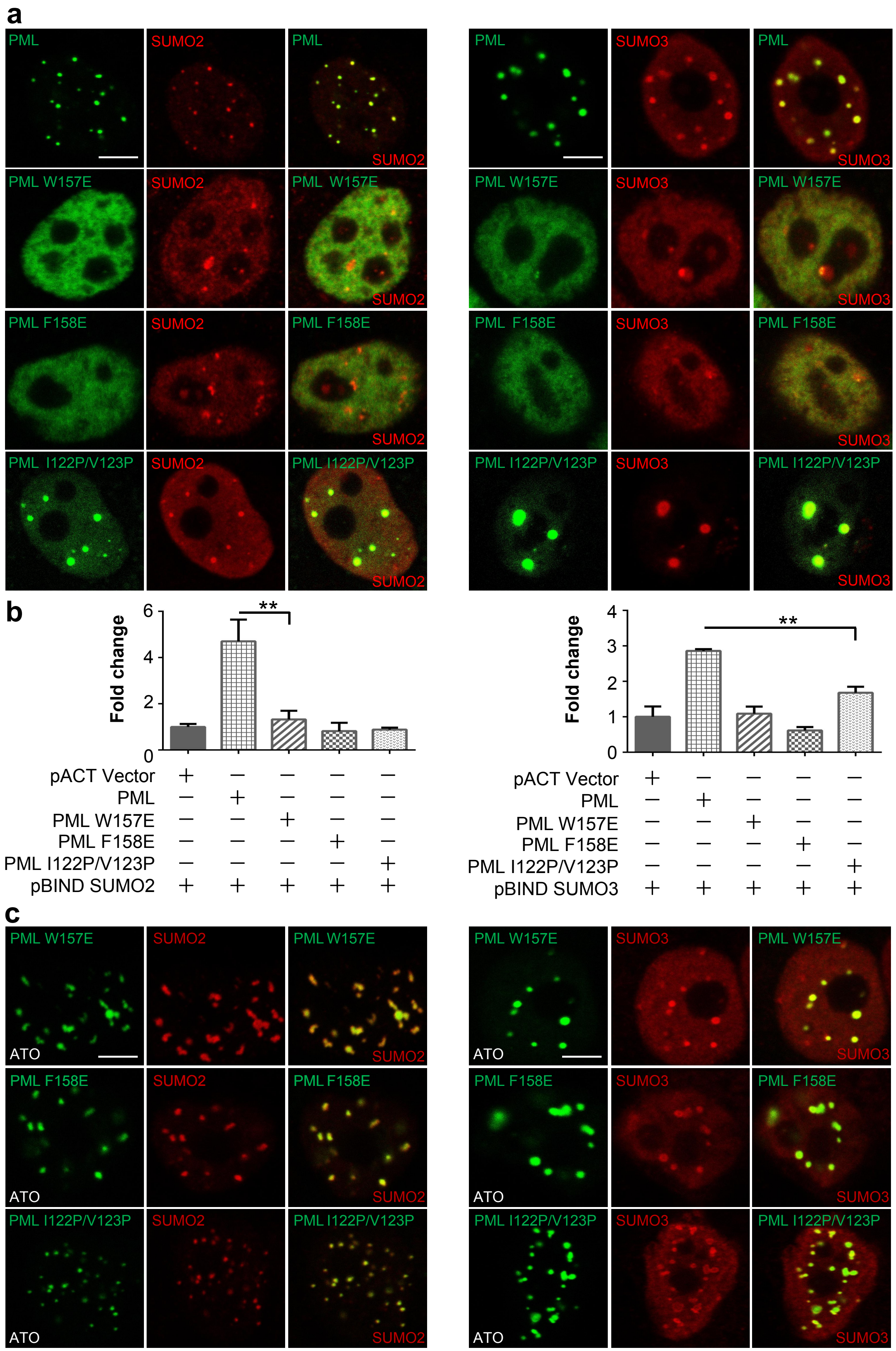

Supplementary Figure 6. PML oligomerization is required for partners recruitment. a) Immuno co-localizations between WT/mutants PML isoform IV and SUMO2/3. Scar bar is 5 μm. b) Mammalian two-hybrid characterization between WT/mutants PML isoform IV and SUMO2/3. The interactions between WT/mutants and SUMO2/3 are all normalized against the pACT vector:pBIND-SUMO2/3 interaction (=1).Values are means ± S.E. **, $p < 0.01$. Source data are provided as a source Data file. c) ATO treatment and the NB-enhanced SUMO2/3 interaction. Scar bar is 5 μm.

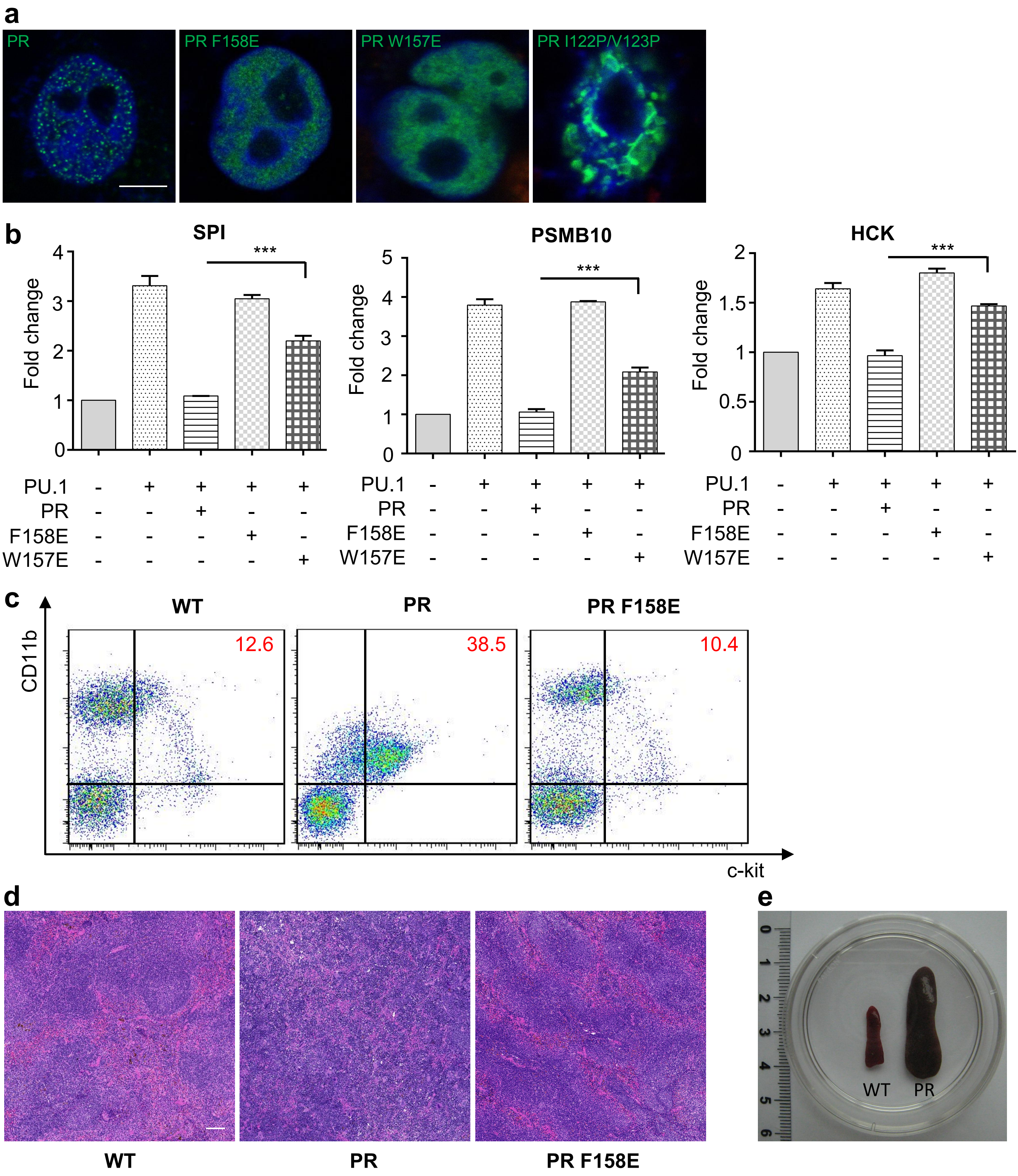

Supplementary Figure 7. **B1 oligomerization in PML-RARα.** a) Speckle formation assay. PML-RARα can oligomerize and form small speckles in nucleoli[3]. This was used to monitor the presence of B1 oligomerization in PML-RARα. The HeLaPml-/- cells expressing WT/mutants PML-RARα (green) were visualized by immunofluorescence. Scar bar is 5 μm. b) B1 oligomerization is important for the transcription activity of PML-RARα. As reported, PR could repress the transcription activity of PU.1 as monitored by luciferase assay[4]. Here, the plasmids pGL3-basic, pLVX-IRES-PR/mutants, pCMV4-PU.1 and pRL-SV40 were all co-transfected to 293T cells, followed by the examination of luciferase activities. Source data are provided as a Source Data file. The results are all normalized against the blank (=1). Values are means ± S.E. ***, $p < 0.001$. c-e) Standard checks such as flow cytometry analysis of c-Kit, Gr-1 and Mac-1 in bone marrow cells (c) HE staining of spleen cells (d) and visual inspection of spleens from WT and PR mice (e) were used to confirm APL development. Scar bar is 100 μm.

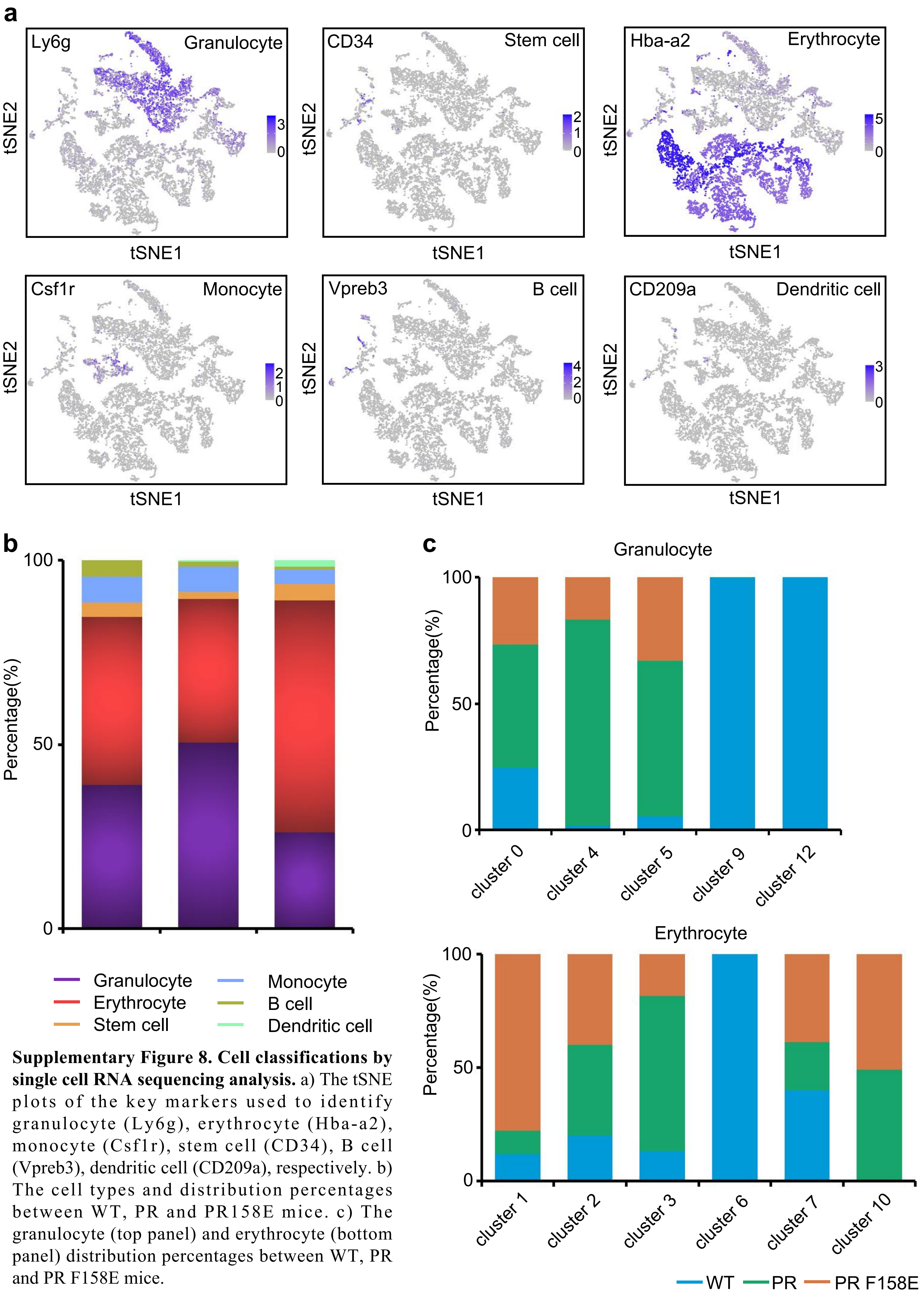

Supplementary Figure 8. Cell classifications by single cell RNA sequencing analysis. a) The tSNE plots of the key markers used to identify granulocyte (Ly6g), erythrocyte (Hba-a2), monocyte (Csf1r), stem cell (CD34), B cell (Vpreb3), dendritic cell (CD209a), respectively. b) The cell types and distribution percentages between WT, PR and PR158E mice. c) The granulocyte (top panel) and erythrocyte (bottom panel) distribution percentages between WT, PR and PR F158E mice.

Supplementary Table 1. Single cell RNA sequencing statistics.

	WT	PR	F158E
Platform	Novaseq	Novaseq	Novaseq
Raw Bases(G)	220.88	227.3	263.21
Number of cells identified by Cell Ranger	4,161	5,588	4,578
Median reads per cell identified by Cell Ranger	176,941	135,581	191,641
Median genes per cell identified by Cell Ranger	917	1,167	824
The number of Cells filtered by Seurat	3,816	4,806	3,874

Supplementary Table 2. The differential expression analysis between PR and PR F158E datastes.

ID	gene_name	log2FoldChange (PR/WT)	pvalue	padj	log2FoldChange (PR F158/PR)	pvalue	padj	Cell type
ENSMUSG00000025491	Ifitm1	3.885193344	7.71E-102	9.33E-99	-4.513845317	7.96E-135	1.71E-131	granulocyte
ENSMUSG00000071561	BC100530	2.670720471	1.43E-38	3.47E-36	-4.183866087	1.85E-82	1.99E-79	granulocyte
ENSMUSG00000054905	Stfa3	2.477749852	2.74E-26	3.28E-24	-2.398900362	1.04E-24	1.58E-22	granulocyte
ENSMUSG00000022902	Stfa2	2.004173799	3.17E-22	3.11E-20	-2.109544206	7.73E-28	1.39E-25	granulocyte
ENSMUSG00000069792	Wfdc17	1.983160914	1.70E-45	5.60E-43	-2.937443633	2.53E-91	3.40E-88	granulocyte
ENSMUSG00000060591	Ifitm2	1.770919488	2.15E-257	2.34E-253	-2.122857709	0	0	granulocyte
ENSMUSG00000030069	Prok2	1.609873874	3.44E-22	3.34E-20	-1.845714401	3.04E-31	6.30E-29	granulocyte
ENSMUSG00000021728	Emb	1.503291755	3.67E-43	1.11E-40	-1.5292766	1.09E-44	3.57E-42	granulocyte
ENSMUSG00000041736	Tspo	1.78327406	3.33E-16	1.11E-12	-1.865136283	1.74E-145	8.97E-142	granulocyte
ENSMUSG00000094777	Hist1h2ap	2.401121774	1.72E-14	5.06E-12	-2.8035863	2.52E-90	4.14E-87	granulocyte
ENSMUSG00000025492	Ifitm3	2.00341661	2.37E-25	3.30E-22	-2.047254276	1.80E-122	6.91E-119	granulocyte
ENSMUSG00000027506	Tpd52	1.787448664	3.61E-07	3.05E-05	-1.856805198	4.29E-35	1.98E-32	granulocyte
ENSMUSG00000039109	F13a1	1.712528528	0.000702377	0.016415308	-2.054791129	3.95E-23	1.03E-20	granulocyte
ENSMUSG00000069917	Hba-a2	-1.709478133	3.84E-29	5.73E-27	2.77689981	4.04E-20	7.30E-18	granulocyte
ENSMUSG00000052305	Hbb-bs	-2.405036701	5.45E-10	5.44E-07	2.609024809	1.45E-63	1.36E-60	granulocyte
ENSMUSG00000069919	Hba-a1	-2.547301507	1.33E-09	1.11E-06	2.926450625	1.26E-66	1.44E-63	granulocyte
ENSMUSG00000073940	Hbb-bt	-2.723331658	3.16E-12	4.51E-09	2.733606747	5.00E-64	5.14E-61	granulocyte
ENSMUSG00000001865	Cpa3	2.802588932	3.86E-08	6.31E-06	-2.402530581	3.72E-06	0.0003908	stem cell
ENSMUSG00000059108	Ifitm6	2.780293019	8.37E-11	2.77E-08	-3.098814413	3.56E-11	1.82E-08	stem cell
ENSMUSG00000024681	Ms4a3	2.643377038	5.95E-13	3.29E-10	-2.696302291	1.07E-14	1.16E-11	stem cell

ENSMUSG00000069792	Wfdc17	2.577206467	2.05E-11	7.67E-09	-3.65622867	2.97E-22	8.73E-19	stem cell
ENSMUSG00000040314	Ctsg	2.551281812	9.72E-11	3.13E-08	-2.493057243	6.43E-09	1.58E-06	stem cell
ENSMUSG00000094777	Hist1h2ap	2.478797609	2.31E-12	1.07E-09	-1.538363928	9.96E-06	0.0008814	stem cell
ENSMUSG00000009350	Mpo	2.252413443	2.25E-10	6.69E-08	-2.844491627	1.09E-12	8.00E-10	stem cell
ENSMUSG00000031722	Hp	2.189871503	3.40E-14	2.63E-11	-2.550197627	5.73E-14	5.62E-11	stem cell
ENSMUSG00000044734	Serpinb1a	2.085117228	1.20E-12	5.81E-10	-1.722800789	6.93E-11	3.40E-08	stem cell
ENSMUSG00000037095	Lrg1	2.048458351	4.19E-09	9.18E-07	-2.64386573	1.08E-14	1.16E-11	stem cell
ENSMUSG00000060063	Alox5ap	1.936634615	7.58E-14	5.17E-11	-1.851602297	2.20E-11	1.18E-08	stem cell
ENSMUSG00000024680	Ms4a2	1.915969798	5.93E-05	0.003029744	-1.966278238	4.26E-05	0.0028320	stem cell
ENSMUSG00000079419	Ms4a6c	1.9092165	2.40E-08	4.14E-06	-2.421765478	1.57E-11	8.78E-09	stem cell
ENSMUSG00000025351	Cd63	1.82977122	9.07E-08	1.30E-05	-1.977964155	5.44E-08	1.03E-05	stem cell
ENSMUSG00000024659	Anxa1	1.812351442	7.89E-13	4.16E-10	-1.802467147	7.41E-10	2.36E-07	stem cell
ENSMUSG00000024480	Ap3s1	1.773313222	4.69E-15	4.53E-12	-1.62206947	1.02E-13	9.25E-11	stem cell
ENSMUSG00000031584	Gsr	1.76547598	2.27E-13	1.39E-10	-1.687508112	1.24E-11	7.53E-09	stem cell
ENSMUSG00000068129	Cst7	1.750094624	3.15E-10	9.15E-08	-1.59228737	3.92E-09	1.02E-06	stem cell
ENSMUSG00000051748	Wfdc21	1.718010694	2.62E-09	6.08E-07	-2.133362652	5.14E-09	1.31E-06	stem cell
ENSMUSG00000071713	Csf2rb	1.676518257	2.49E-07	3.15E-05	-1.970107158	2.47E-10	9.40E-08	stem cell
ENSMUSG00000071714	Csf2rb2	1.62924433	4.65E-07	5.68E-05	-2.074417474	1.23E-12	8.55E-10	stem cell
ENSMUSG00000027360	Hdc	1.535590417	0.000270656	0.009973026	-2.185946347	7.53E-09	1.77E-06	stem cell
ENSMUSG00000029373	Pf4	-3.291541276	3.28E-09	7.33E-07	2.790263433	4.57E-05	0.0029912	stem cell
ENSMUSG00000030717	Nupr1	3.150049785	4.54E-60	8.70E-57	-3.142231689	1.31E-104	3.14E-101	erthrocyte
ENSMUSG00000032715	Trib3	2.976225	2.65E-54	3.62E-51	-2.920205948	1.23E-93	1.31E-90	erthrocyte
ENSMUSG00000035692	Isg15	2.931058182	1.56E-43	1.36E-40	-1.70276804	3.82E-25	2.53E-23	erthrocyte
ENSMUSG00000038539	Atf5	2.678075912	5.85E-46	7.00E-43	-3.024418423	8.13E-96	1.11E-92	erthrocyte
ENSMUSG00000032578	Cish	2.5089803	2.90E-45	3.08E-42	-2.288976461	1.02E-68	4.65E-66	erthrocyte

ENSMUSG00000025408	Ddit3	2.300177757	2.74E-38	1.75E-35	-2.657505765	1.25E-82	8.57E-80	erthrocyte
ENSMUSG00000005667	Mthfd2	2.114008622	2.22E-41	1.77E-38	-2.577457317	1.70E-92	1.63E-89	erthrocyte
ENSMUSG00000027806	Tsc22d2	2.033068336	5.13E-36	2.89E-33	-1.957685573	5.52E-56	1.51E-53	erthrocyte
ENSMUSG00000025491	Ifitm1	2.010645978	4.27E-29	1.41E-26	-3.118584175	8.25E-95	9.89E-92	erthrocyte
ENSMUSG00000032026	Rexo2	1.894651401	1.05E-33	4.59E-31	-1.754998167	1.07E-50	2.28E-48	erthrocyte
ENSMUSG00000029752	Asns	1.84899469	4.40E-32	1.76E-29	-2.610935899	9.40E-91	7.51E-88	erthrocyte
ENSMUSG00000106438	Gm32051	1.777406372	5.36E-23	1.12E-20	-2.003165618	1.61E-48	3.15E-46	erthrocyte
ENSMUSG00000031490	Eif4ebp1	1.741651626	1.24E-25	3.11E-23	-2.425545013	2.17E-70	1.10E-67	erthrocyte
ENSMUSG00000068739	Sars	1.736043646	4.16E-29	1.41E-26	-2.125371636	1.33E-66	5.55E-64	erthrocyte
ENSMUSG00000030738	Eif3c	1.721240382	1.33E-28	4.23E-26	-2.064825539	5.28E-62	1.95E-59	erthrocyte
ENSMUSG00000039745	Htatip2	1.686455764	9.07E-44	8.69E-41	-1.555069463	3.58E-63	1.37E-60	erthrocyte
ENSMUSG00000025403	Shmt2	1.549551428	3.30E-19	5.45E-17	-2.043012708	9.71E-50	2.02E-47	erthrocyte
ENSMUSG00000009013	Dynll1	1.542181346	2.66E-32	1.11E-29	-1.587641906	7.69E-53	1.75E-50	erthrocyte
ENSMUSG00000047443	Erfe	2.477109077	1.03E-48	6.43E-46	-1.808256852	3.20E-25	6.01E-23	erthrocyte
ENSMUSG00000069792	Wfdc17	2.176378291	5.49E-24	8.60E-22	-3.550349373	1.25E-54	6.76E-52	erthrocyte
ENSMUSG00000031762	Mt2	2.077762101	5.30E-16	4.00E-14	-2.174167156	3.58E-17	3.46E-15	erthrocyte
ENSMUSG00000050747	Trim15	2.071458455	3.41E-30	9.78E-28	-1.874551606	4.66E-25	8.57E-23	erthrocyte
ENSMUSG00000074269	Rec114	-1.951412604	2.29E-29	8.12E-27	1.776919017	1.62E-33	1.65E-31	erthrocyte

Supplementary Table 3. Primers used for sub-cloning.

Primer Name	Primer Sequence(5' to 3')
pET32a-PML-B1 box-F	cgggatcccggcagattgtggatgcgcaggctg
pET32a-PML-B1 box-R	ccgctcgagtcactctgctaggggccgggcctc
pET15b-PML1-256-F	gggaattccatatggagcctgcacccgcccgat
pET15b-PML1-256-R	ccgctcgagctacgcgccaaaggcactatcctg
pET15b-NcoI-SUMO-F	aactttaagaaggagatataccatgggtcatcaccatcatcat
pET15b-NcoI-SUMO-R	atgatgatgatggctgctgcccatggcaatctgttctctgtgagc
pACT-PML-F	cgggatccgtgagcctgcacccgcccgatctc
pACT-PML-R	gctctagactaaattagaaaggggtgggggtag
pBIND-PML-F	cgggatccgtgagcctgcacccgcccgatctc
pBIND-PML-R	gctctagactaaattagaaaggggtgggggtag
pEGFP-C1-PML-F	ggaattcaatggagcctgcacccgcccgatc
pEGFP-C1-PML-R	cgggatccctaaattagaaaggggtgggggtag
pLVX-HA-PML-F	ggaattcgccaccatgtacccatacgatgttccagattacgctgagcctgcacccg
pLVX-HA-PML-R	cgggatccctaaattagaaaggggtgggggtag
pEGFP-C1-PML_I-F	ggaattcaatggagcctgcacccgcccgatctc
pEGFP-C1-PML_I-R	cgggatcctcagctctgctgggaggccctctc
pFlAG-CMV4-DAXX-F	ggaattcaatggccaccgctaacagcatcatc
pFlAG-CMV4-DAXX-R	cgggatccctaatcagagtctgagagcacgatg
pFlAG-CMV4-SUMO2-F	ggaattcagtgccgacgaaaagcccaag
pFlAG-CMV4-SUMO2-R	cgggatcctcagtagacacctcccgtctgctg
pFlAG-CMV4-SUMO3-F	ggaattcagttccgaggagaagcccaag
pFlAG-CMV4-SUMO3-R	cgggatccctagaaactgtgccctgccag
pBIND-DAXX-F	cgggatccgtatggccaccgctaacagcatcatc
pBIND-DAXX-R	gctctagactaatcagagtctgagagcacgatg
pBIND-SUMO2-F	cgggatccgtgccgacgaaaagcccaag
pBIND-SUMO2-R	gctctagatcagtagacacctcccgtctgctg
pBIND-SUMO3-F	cgggatccgttccgaggagaagcccaag
pBIND-SUMO3-R	gctctagactagaaactgtgccctgccag

PML, PML isform IV; PML_I, PML isform I.

Supplementary Table 4. Primers used for mutagenesis.

Primer Name	Primer Sequence(5' to 3')
PML-W157A-F	ctcgtgcttgaggaacgcctggtgtgcctcgaag
PML-W157A-R	cttcgaggcacaccaggcgttcctcaagcacgag
PML-W157E-F	ctcgtgcttgaggaactcctggtgtgcctcgaag
PML-W157E-R	cttcgaggcacaccaggagttcctcaagcacgag
PML-W157K-F	ctcgtgcttgaggaacttctggtgtgcctcgaag
PML-W157K-R	cttcgaggcacaccagaagttcctcaagcacgag
PML-F158A-F	gcctcgtgcttgagggcccactggtgtgcctc
PML-F158A-R	gaggcacaccagtgggccctcaagcacgaggc
PML-F158E-F	ggcctcgtgcttgagctcccactggtgtgcctc
PML-F158E-R	gaggcacaccagtgggagctcaagcacgaggcc
PML-F158K-F	ggcctcgtgcttgagcttccactggtgtgcctc
PML-F158K-R	gaggcacaccagtggaagctcaagcacgaggcc
PML-W157F158E-F	ccgggcctcgtgcttgagctcctcctggtgtgcctcgaagca
PML-W157F158E-R	tgcttcgaggcacaccaggaggagctcaagcacgaggcccgg
PML-I122V123P-F	acacagcctgcgcatccggaggctgccggtacaccgaca
PML-I122V123P-R	tgtcggtgtaccggcagcctccggatgcgcaggctgtgt

Supplementary Table 5. Data-collection and scattering-derived parameters.

**Data-collection parameters**			
Instrument	BL19U2 Beamline		
Wavelength (Å)	1.03		
q range ($Å^{-1}$)	0.01-0.25		
Exposure time (sec)	1		
Concentration range (mg ml^{-1})	1-10		
Temperature (K)	298		
**Structural parameters**			
Proteins	B1 WT	B1 W157E	B1 F158E
I (0) [from Guinier]	16.09 ± 0.025	7.96 ± 0.014	11.14 ± 0.025
Rg (Å) [from Guinier]	15.86 ± 0.11	12.92 ± 0.28	15.28 ± 0.06
Dmax (Å)	71.50	49.68	65.58
**Software employed**			
Primary data reduction	PRIMUS		
Data processing	PRIMUS/GNOM		
Computation of model intensities	CRYSOL		
Estimation of oligomers in solution	OLIGOMER		

Rg: Radius of Gyration, Dmax: maximum particle size

REFERENCES

1. Lallemand-Breitenbach V*, et al.* Role of Promyelocytic Leukemia (PML) Sumolation in Nuclear Body Formation, 11S Proteasome Recruitment, and As(2)O(3)-induced PML or PML/Retinoic Acid Receptor alpha Degradation. *J. Exp. Med.* **193**, 1361-1372. (2001).

2. Huang SY*, et al.* The B-box 1 dimer of human promyelocytic leukemia protein. *J. Biol. NMR* **60**, 275-281 (2014).

3. Dyck JA, Warrell RP, Jr., Evans RM, Miller WH, Jr. Rapid diagnosis of acute promyelocytic leukemia by immunohistochemical localization of PML/RAR-alpha protein. *Blood* **86**, 862-867 (1995).

4. Wang K*, et al.* PML/RARalpha Targets Promoter Regions Containing PU.1 Consensus and RARE Half Sites in Acute Promyelocytic Leukemia. *Cancer Cell* **17**, 186-197 (2010).
