# Supplementary material for: B1 oligomerization regulates PML nuclear body biogenesis and leukemogenesis
Source: Nat Commun. 2019 Aug 22;10:3789. doi: 10.1038/s41467-019-11746-0 (PMC6706441; doi:10.1038/s41467-019-11746-0)
Supplement: Supplementary file 3 — Reporting Summary [file 41467_2019_11746_MOESM3_ESM.pdf]

nature research

Corresponding author(s): GUOYU MENG

Last updated by author(s): Jul 24, 2019

Reporting Summary

Nature Research wishes to improve the reproducibility of the work that we publish. This form provides structure for consistency and transparency in reporting. For further information on Nature Research policies, see Authors & Referees and the Editorial Policy Checklist.

Statistics

For all statistical analyses, confirm that the following items are present in the figure legend, table legend, main text, or Methods section.

n/a	Confirmed	
☐	☒	The exact sample size (*n*) for each experimental group/condition, given as a discrete number and unit of measurement
☐	☒	A statement on whether measurements were taken from distinct samples or whether the same sample was measured repeatedly
☐	☒	The statistical test(s) used AND whether they are one- or two-sided<br>*Only common tests should be described solely by name; describe more complex techniques in the Methods section.*
☒	☐	A description of all covariates tested
☒	☐	A description of any assumptions or corrections, such as tests of normality and adjustment for multiple comparisons
☐	☒	A full description of the statistical parameters including central tendency (e.g. means) or other basic estimates (e.g. regression coefficient) AND variation (e.g. standard deviation) or associated estimates of uncertainty (e.g. confidence intervals)
☐	☒	For null hypothesis testing, the test statistic (e.g. *F*, *t*, *r*) with confidence intervals, effect sizes, degrees of freedom and *P* value noted<br>*Give P values as exact values whenever suitable.*
☒	☐	For Bayesian analysis, information on the choice of priors and Markov chain Monte Carlo settings
☒	☐	For hierarchical and complex designs, identification of the appropriate level for tests and full reporting of outcomes
☒	☐	Estimates of effect sizes (e.g. Cohen's *d*, Pearson's *r*), indicating how they were calculated

Our web collection on statistics for biologists contains articles on many of the points above.

Software and code

Policy information about availability of computer code

Data collection	GraphPad Prism software;<br>Origin8 software;<br>Cells were imaged on a Zeiss confocal microscope, using TSP SP8, Leica<br>For western blot analysis we used ImageQuant LAS-4000 software from FujiFilm<br>For crystallography we used CCP4 softwares and Phenix.refine.<br>For SAXS we used PRIMUS and CRYSOL.<br>For CD analysis we used CDNN.<br>For analytical ultracentrifugation we used SEDFIT.<br>For single cell RNA sequencing we used Cell Ranger and Seurat.
Data analysis	the analysis was used through the softwares mentioned above

For manuscripts utilizing custom algorithms or software that are central to the research but not yet described in published literature, software must be made available to editors/reviewers. We strongly encourage code deposition in a community repository (e.g. GitHub). See the Nature Research guidelines for submitting code & software for further information.

Data

Policy information about availability of data

All manuscripts must include a data availability statement. This statement should provide the following information, where applicable:

- Accession codes, unique identifiers, or web links for publicly available datasets
- A list of figures that have associated raw data
- A description of any restrictions on data availability

Coordinates and structure factors have been deposited under accession code 6IMQ [https://www.rcsb.org/structure/6IMQ]. The source data underlying Figs 1cd,

4a-c, 4ef, 5a–c, 5ef, 6b, 7a, c, and Supplementary Figs 3b, 4a, 4c, 4e, 5b, 6b and 7b are provided as a Source Data file. Other data are available from the corresponding author upon reasonable request.

Field-specific reporting

Please select the one below that is the best fit for your research. If you are not sure, read the appropriate sections before making your selection.

☒ Life sciences ☐ Behavioural & social sciences ☐ Ecological, evolutionary & environmental sciences

For a reference copy of the document with all sections, see nature.com/documents/nr-reporting-summary-flat.pdf

Life sciences study design

All studies must disclose on these points even when the disclosure is negative.

Sample size	We use PML-RARa and PML-RARa F158E (PR F158E) transgenic mice at the same age (i.e. 78 weeks). And three mouse for single cell analysis.
Data exclusions	all the values were included.
Replication	there was a triplicate for each experiment at least as described in the legends for each figure.
Randomization	all samples collected were used for this study without any discrimination.
Blinding	The investigators were not blinded to allocation during experiments and outcome assessment.

Reporting for specific materials, systems and methods

We require information from authors about some types of materials, experimental systems and methods used in many studies. Here, indicate whether each material, system or method listed is relevant to your study. If you are not sure if a list item applies to your research, read the appropriate section before selecting a response.

Materials & experimental systems

n/a	Involved in the study	
☐	☒	Antibodies
☐	☒	Eukaryotic cell lines
☒	☐	Palaeontology
☐	☒	Animals and other organisms
☒	☐	Human research participants
☒	☐	Clinical data

Methods

n/a	Involved in the study	
☒	☐	ChIP-seq
☐	☒	Flow cytometry
☒	☐	MRI-based neuroimaging

Antibodies

Antibodies used	anti-HA tag (Abcam cat# ab9110) , anti-actin (Senta Cruz , cat# sc47778), anti-PML (Abcam, cat# ab72137), anti-Mouse (Cell Signaling Technology, cat# 7076P2), anti-Rabbit (Cell Signaling Technology cat# 7074P2),Alexa Fluor 568 conjugate anti-Mouse (Invitrogen, Product # A-11031), Monoclonal anti-Flag (Sigma, cat# F1804-50UG)
Validation	following manufacturer validation

Eukaryotic cell lines

Policy information about cell lines

Cell line source(s)	Hela cells; 293T cells
Authentication	Cell lines were obtained from original sources and were not further authenticated.
Mycoplasma contamination	Cells were regularly tested to ensure none mycoplasma contamination.
Commonly misidentified lines (See ICLAC register)	N/A

Animals and other organisms

Policy information about studies involving animals; ARRIVE guidelines recommended for reporting animal research

Laboratory animals	study involved animals (mice) as stated in methods
Wild animals	N/A
Field-collected samples	no samples were collected in Field
Ethics oversight	All animal experiments and care were carried out as approved by the Animal Care and Use Committee at Experimental Animal Centre in Shanghai Jiao Tong University School of Medicine, China.

Note that full information on the approval of the study protocol must also be provided in the manuscript.

Flow Cytometry

Plots

Confirm that:

☒ The axis labels state the marker and fluorochrome used (e.g. CD4-FITC).

☒ The axis scales are clearly visible. Include numbers along axes only for bottom left plot of group (a 'group' is an analysis of identical markers).

☒ All plots are contour plots with outliers or pseudocolor plots.

☒ A numerical value for number of cells or percentage (with statistics) is provided.

Methodology

Sample preparation	We get cells from bone marrow or spleen to identify transgenic mouse pathogenesis.
Instrument	flow cytometry (LSR2,BD)
Software	flowjo software
Cell population abundance	> 10,000 cells per experiment were analyzed
Gating strategy	FSC-A/SSC-A gates of the starting cell population were used to dicriminate between viable cells and cells deribris. Singlet and doublet cells were discriminated using FSC-A/FSC-W gating. The CD11b and c-kit gates were set using related sample without fluorochrome as a control.

☒ Tick this box to confirm that a figure exemplifying the gating strategy is provided in the Supplementary Information.
